# Sleeping site ecology, but not sex, affect ecto- and hemoparasite risk, in sympatric, arboreal primates (*Avahi occidentalis* and *Lepilemur edwardsi*)

**DOI:** 10.1186/s12983-017-0228-7

**Published:** 2017-09-20

**Authors:** May Hokan, Christina Strube, Ute Radespiel, Elke Zimmermann

**Affiliations:** 1 Institute of Zoology, University of Veterinary Medicine Hannover, Buenteweg 17, 30559 Hannover, Germany; 2 Institute for Parasitology, Centre for Infection Medicine, University of Veterinary Medicine Hannover, Buenteweg 17, 30559 Hannover, Germany

**Keywords:** Parasite, Mites, Ticks, Microfilaria, Seasonality, Sociality, Sex, Ecology, Behavior, Western woolly lemur, Milne-Edward’s sportive lemur, Primate, Tropics, Madagascar

## Abstract

**Background:**

A central question in evolutionary parasitology is to what extent ecology impacts patterns of parasitism in wild host populations. In this study, we aim to disentangle factors influencing the risk of parasite exposure by exploring the impact of sleeping site ecology on infection with ectoparasites and vector-borne hemoparasites in two sympatric primates endemic to Madagascar. Both species live in the same dry deciduous forest of northwestern Madagascar and cope with the same climatic constraints, they are arboreal, nocturnal, cat-sized and pair-living but differ prominently in sleeping site ecology. The Western woolly lemur (*Avahi occidentalis*) sleeps on open branches and frequently changes sleeping sites, whereas the Milne-Edward’s sportive lemur (*Lepilemur edwardsi*) uses tree holes, displaying strong sleeping site fidelity. Sleeping in tree holes should confer protection from mosquito-borne hemoparasites, but should enhance the risk for ectoparasite infestation with mites and nest-adapted ticks. Sex may affect parasite risk in both species comparably, with males bearing a higher risk than females due to an immunosuppressive effect of higher testosterone levels in males or to sex-specific behavior. To explore these hypotheses, ectoparasites and blood samples were collected from 22 individuals of *A. occidentalis* and 26 individuals of *L. edwardsi* during the dry and rainy season.

**Results:**

*L. edwardsi,* but not *A. occidentalis*, harbored ectoparasites, namely ticks (*Haemaphysalis lemuris* [Ixodidae], *Ornithodoros* sp. [Argasidae]) and mites (*Aetholaelaps trilyssa*, [Laelapidae]), suggesting that sleeping in tree holes promotes infestation with ectoparasites. Interestingly, ectoparasites were found solely in the hot, rainy season with a prevalence of 75% (*N* = 16 animals). Blood smears were screened for the presence and infection intensity of hemoparasites. Microfilariae were detected in both species. Morphological characteristics suggested that each lemur species harbored two different filarial species. Prevalence of microfilarial infection was significantly lower in *L. edwardsi* than in *A. occidentalis.* No significant difference in infection intensity between the two host species, and no effect of season, daytime of sampling or sex on prevalence or infection intensity was found. In neither host species, parasite infection showed an influence on body weight as an indicator for body condition.

**Conclusions:**

Our findings support that sleeping site ecology affects ectoparasite infestation in nocturnal, arboreal mammalian hosts in the tropics, whereas there is no significant effect of host sex. The influence of sleeping site ecology to vector-borne hemoparasite risk is less pronounced. The observed parasite infections did not affect body condition and thus may be of minor importance for shaping reproductive fitness. Findings provide first evidence for the specific relevance of sleeping site ecology on parasitism in arboreal and social mammals. Further, our results increase the sparse knowledge on ecological drivers of primate host-parasite interactions and transmission pathways in natural tropical environments.

**Electronic supplementary material:**

The online version of this article (10.1186/s12983-017-0228-7) contains supplementary material, which is available to authorized users.

## Background

The distribution and abundance of parasites are influenced, amongst others, by environmental factors as well as interactions with the host’s behavioral ecology. Environmental factors like temperature and rainfall impact patterns of parasitism in the way that warmth and humidity favor hatching of arthropod eggs, usually resulting in higher abundance of temporary ectoparasites and insect vectors, such as mosquitos, in the hot and rainy season [1, 2]. The cattle tick (*Amblyomma variegatum*) becomes more active in the early wet season, when temperature increases [3]. However, in a rainforest-dwelling lemur species, the diademed sifaka (*Propithecus diadema*), ticks (*Haemaphysalis lemuris*) were found to be more prevalent in the dry than in the rainy season [4]. Other etcoparasite infestations do not differ with season. Prevalence of mites (*Spelaeorhynchus praecursor*) and argasid ticks (*Ornithodores* sp.) in bats, for example, did not vary seasonally [5]. Hence, the influence of environmental factors, such as season can vary between the different ectoparasite genera and must therefore be taken into account in studies on parasite infections.

Host behavioral ecology is also described to affect the distribution and abundance of parasites in mammals. Parasite avoidance behaviors, such as auto- and allogrooming as well as mud baths are suggested to reduce ectoparasite infestation, while defecating outside nests or dens may reduce exposure to endoparasites [6]. In a wide range of animals, social grouping is documented to not only provide protection from predators but also from flying insects, such as flies and mosquitoes by reducing exposure of the animal’s body surface [7]. Furthermore, behaviors related to sleeping site ecology are proposed to reduce exposure to insects such as mosquitoes (e.g. *Anopheles* spp.) and the parasites they may transmit. For example, chimpanzees (*Pan troglodytes schweinfurthii*) prefer to build their nests in a tree species (*Cynometra alexandri*) with insect repellant properties, potentially reducing the risk of malaria infection via mosquito bites [8]. Moreover, sleeping in burrows or holes may provide protection from flying insects [9, 10], in addition to conferring essential benefits such as insulation from unfavorable climate conditions or protection from predators [11–13]. On the other hand, burrows of rodents, for instance, provide an excellent habitat for ectoparasites such as mites, fleas or ticks due to their stable, dark, moist, and warm microclimate. For example, fleas and mites co-occur more often in voles (*Microtus* spp.) using deep and complex burrow systems than in a congeneric species, which sleeps above ground or uses shallow burrows [14]. In addition, the year-round presence of the host in such burrows provides ectoparasites with a regular food supply [15, 16]. In bats, roosting habits have been related to prevalence and species richness of a specific bat ectoparasite, the bat fly, with heavier parasitism found in bats using more permanent, enclosed roosts [17]. Ectoparasite infestation may therefore constitute an important cost of sleeping in regularly revisited, confined spaces. These ectoparasites, especially the flying insects, may function as vectors transmitting hemoparasites such as *Plasmodium* spp., *Babesia* spp. or filarial nematodes. The first two are haemosporidian parasites, that can be detected by microscopy and differentiated by comparing shape and size of the parasitic stages located inside the erythrocytes as well as size and position of their nucleus [18, 19]. Also microfilaria can be well detected by microscopy, situated between erythrocytes. Species can be differentiated using morphological parameter such as body length, size and proportion of the cephalic space, position of the nerve ring and form of the tail [20, 21].

Furthermore, host traits such as sex and body mass can affect patterns of parasitism. Male flying squirrels have been reported to be more susceptible to ecto- and hemoparasites than females [22], which may be due to an immunosuppressive effect of testosterone or to sex-specific differences in behavior [23]. Nevertheless, a number of studies found no sex differences in prevalence or infection intensity of parasites [24, 25] and some even found higher parasite infection rates in females [26]. Body condition, measured by body mass, is discussed to be linked to parasite infection and ultimately may affect fitness [27–29]. In monkeys and apes, animals in a poorer condition are often more heavily parasitized than indivudals in better condition [30]. In contrast, in rufous mouse lemurs (*Microcebus rufus*) it was found that individuals bearing a higher ecto- and endoparasite load had a better body condition [31]. Thus, the evidence for an influence of sex and body mass on parasite infection in wild hosts is ambiguous and requires further attention.

The goal of this study was to evaluate the impact of host sleeping site ecology, season and sex on patterns of ecto- and hemoparasite infections in a tropical seasonal environment, using two Malagasy sympatric nocturnal primate species as models. The Western woolly lemur (*Avahi occidentalis*) and the Milne Edward’s sportive lemur (*Lepilemur edwardsi*) are both arboreal, dry deciduous forest-dwelling, folivorous primates, endemic to northwestern Madagascar [32]. Both species are nocturnal, exhibit a comparable body mass of approximately 1 kg with no sex dimorphism, share the same habitat and thereby same climate conditions and are pair-living and thus match in social pattern [33, 34]. However, they differ prominently in their choice of sleeping sites as well as in their sleeping site related behavior: *A. occidentalis* sleeps on open branches or tree forks and changes its sleeping sites frequently, whereas *L. edwardsi* sleeps in tree holes with high sleeping site fidelity [35, 36]. Thus, these two host species present unique models to assess the effect of host sleeping site ecology on parasite risk, while controlling for the factors climate condition, activity, host body size and sociality. The following hypotheses were explored: Ectoparasite and hemoparasite prevalence, infection intensity and species richness are assumed to be higher in the host species sleeping in tree holes. In addition, both ecto- and hemoparasites are assumed to be more prevalent during the warm, rainy season and more prevalent in males than in females. As an indicator for the host’s condition and thereby fitness, we examined the effect of parasitism on host’s body mass.

## Methods

### Study site

**Fig. 1:**
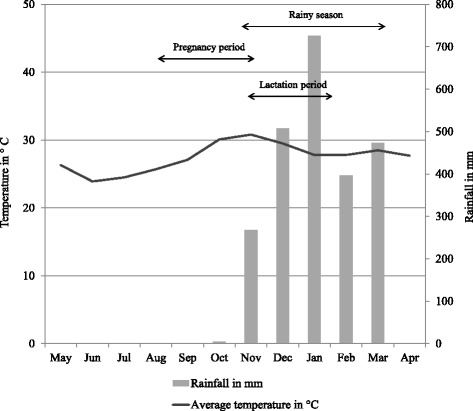
Climate chart showing temperature and rainfall at Ankarafantsika National Park May 2013–April 2014

The study was conducted in a 30.6 ha forest parcel named Jardin Botanique A (JBA), located at 16° 19′ S, 46° 48′ E in the Ankarafantsika National Park in northwestern Madagascar. The park consists of dry deciduous forest and is subject to pronounced seasonality, with a dry season from May to October and a hot, rainy season from November to March (Fig. 1).

### Sample collection

Sample collection took place during two periods, from July to October 2013 and from March to May 2014, representing the dry and rainy season, respectively. Twenty-two individuals of *Avahi occidentalis* (10 male, 12 females) were captured during the study period by remote immobilization with a combination of ketamine (Ketanest®, 25 mg/ml) and xylazine (Rompun®, 20 mg/ml) using a blowpipe and 1 ml cold air pressure darts (Telinject®, Germany). Seven of these individuals were sampled more than once (Additional file 1). Dosages based on estimated body weights were as follows: 10 mg/kg ketamine and 0.5 mg/kg xylazine. Twenty-six individuals of *Lepilemur edwardsi* (12 males, 14 females) were captured directly in their tree holes and sedated with the same drug combination. Twelve of these individuals were sampled more than once (Additional file 1).

All captured individuals were weighed (5 Kg-balance, AEG, precision: 1 g) and macroscopically examined for ectoparasites. All ticks and representative samples of mites were removed and preserved in 90% ethanol. The body parts of the hosts harboring ectoparasites were noted in a protocol. Blood was taken either from the femoral vein or collected opportunistically during the process of ear marking and tissue collection. In total 29 blood samples (17 in the dry and 12 in the rainy season) from *A. occidentalis* and 44 blood samples (27 in the dry and 17 in the rainy season) from *L. edwardsi* were collected*.* Three to five blood smears per sample were prepared, air dried and fixed with methanol. Additionally, in the rainy season one to three drops of blood from 16 *L. edwardsi* and from 9 *A. occidentalis* were collected into cryotubes with 0.5 ml RNAlater (Qiagen, Hilden, Germany) and frozen at −12 °C.

All procedures were approved by the Ministère de l’Environnement, de l’Ecologie et des Forêts and Madagascar National Parks (MNP) and necessary research permits were obtained from the Malagasy authorities (License N° 167 /13/MEF/SG/DGF/DCB.SAP/SCB obtained on the 13th of July 2013 and N°072/14 obtained on the 12th of March 2014).

### Microscopic examination

Ectoparasites were mounted in polyvinyl lactophenol for morphological identification. Blood smears were Giemsa stained and scanned for the presence of hemoparasites. Ecto- and hemoparasites were morphologically identified. All parasites were photographed with an Olympus CAMEDIA C-5050 Zoom digital camera, then visualized and measured with the cell^B Image Acquisition Software (version 3.1; Olympus Soft Imaging Solutions).

### Quantifying hemoparasitemia

Initially, the exact quantity of blood on each slide was unknown. However, in order to quantify the level of parasitemia, the quantity of blood on each slide was determined. Therefore, all blood smears were photographed with a Canon EOS 60D under the same conditions with a constant distance between the camera lens and the slide (ISO - 100, aperture F/16, shutter speed 1/30 s). The photos were cropped to the same size and showed only the blood slide without margins. They were then transformed into black and white. The color intensity of the whole image was measured with the image-processing program ImageJ (version 1.48; U.S. National Institutes of Health). The same technique was applied to blood smears from baboons prepared as reference with known blood quantities. These quantities varied between 1 and 30 μl augmenting in steps of 1 μl for the smears with volumes from 1 to 10 μl and in steps of 2 μl for the smears with volumes from 10 to 30 μl. Of each quantity, five slides were prepared, resulting in a total of 100 reference slides. The blood was obtained from baboons from the German Primate Center (Göttingen, Germany), representing residual amounts of blood taken for medical examination. The measured intensities of the baboon blood photos allowed assigning each blood amount to an intensity interval. Thus, the amount of blood on each of the lemur blood smears could be determined with an accuracy of ± 1 μl. Microfilaria present on each blood smear were counted and absolute counts were transformed into microfilaria per μl of blood.

### DNA extraction, PCRs and sequence analyses

The RNAlater-preserved blood samples were dissolved with 0.5 ml distilled water and centrifuged at 4000 x *g* for 3 min. After centrifugation, the supernatant containing the RNAlater was removed. For DNA extraction with the NucleoSpin® Tissue kit (Macherey-Nagel, Düren, Germany), 180 μl lysis buffer and 25 μl proteinase K was added to the blood pellet and incubated overnight at 56 °C. The following day, DNA was purified according to the manufacturer’s instructions.

To identify the microfilariae observed in a blood sample of *L. edwardsi*, a PCR was performed amplifying the ITS1–5.8S–ITS2 rDNA region using the primer set NC5 and NC2 [37]. The 50 μl reaction mixture contained 5 μl 10× Taq buffer (5 Prime, Hilden, Germany), 1 μl of 10 mM deoxynucleotide triphosphates, 2 μl of each primer (10 μmol each), 1 μl Taq Polymerase (5 Prime, Hilden, Germany) and 2 μl DNA template. PCR was performed using the peqSTAR thermocycler (Peqlab VWR, Erlangen, Germany) under the following conditions: an initial denaturation at 95 °C for 3 min, 30 cycles of 94 °C for 30 s, 55 °C for 30 s (annealing), 71 °C for 30 s (extension), followed by a final elongation step at 72 °C for 10 min. Positive and negative controls were included. The PCR products were visualized by gel electrophoresis on 1% agarose gels.

The amplified fragment was inserted into the pCR4™4-TOPO® vector and cloned into One Shot® TOP10 chemically competent *E. coli* using TOPO® TA cloning kit for sequencing (Invitrogen, Karlsruhe, Germany). Plasmid DNA was obtained using the NucleoSpin® Plasmid Kit (Macherey-Nagel, Dueren, Germany) following the manufacturer’s recommendations. Afterwards, the insert was sequenced by Sanger sequencing (Seqlab Sequence Laboratories Göttingen). The obtained sequence was analysed using Clone Manager Professional Edition 9 (Sci-Ed Software, Denver, USA) and compared to publicly available sequences using BLAST [38].

### Statistical analyses

To assess the difference in ectoparasite prevalence (= percentage of infected individuals) between the two host species and the two sexes, we used a Fisher’s Exact Test (Statistica 6.1, StatSoft. Inc. Tulsa, USA). Regarding hemoparasites, we first analyzed the overall prevalence of microfilaria infection for *A. occidentalis* and for *L. edwardsi*. For this purpose, each host individual was included once per season and a host was considered positive for a season, if at least one of its blood smears from that period was positive. For all subsequent analyses, the information content of multiple sampling was included. In order to assess the influence of host species, sex, season and time of day, when blood samples were collected, on the probability of microfilariae presence, generalized linear mixed models (GLMMs) were constructed with binomial error structure and logit link function. The models contained the variables “species” (*A. occidentalis*, *L. edwardsi*), “sex” (male, female), “season” (dry, rainy) and “time of day” (morning, noon, afternoon, night) as fixed factors. Animal ID was included as random effect, because 21 individuals (28%) of both species contributed more than one sample. We successively tested the different factors one by one by comparing them to a null model containing only the random factor with the Anova function using the Chi-square distribution for determining the *p*-values. We then tested the full model containing all factors to evaluate their significance. Except when examining the factor host species, all variables were tested separately for the host species *A. occidentalis* and *L. edwardsi* in order to assess whether the observed effect is only present in one or in both species. These analyses were performed in R v.3.2.2 [39] using the package lme4 [40].

Additionally, the influence of the same factors (“species”, “sex”, “season” and “time of day”) on the infection intensity, i.e. the level of microfilaremia (number of microfilariae/μl) in microfilaria-positive samples was determined. This was done with the same fixed and random factors as described above, employing a linear mixed effect model (LMM) using the package nlme [41]. For this purpose, microfilaremia values were log-transformed in order to achieve normality in the distribution.

Furthermore, a LMM was constructed to assess the impact of “species”, “sex” and “season” on the length of microfilariae. Systematic length differences between these subsamples might indicate a difference in filarial species.

Finally, we examined the host’s body mass as an indicator for the host’s condition. Using a Mann-Whitney U Test (IBM SPSS Statistics, version 24), it was tested whether there was a difference in body mass between individuals carrying ecto- and hemoparasites and those which did not.

## Results

### Ectoparasites

No ectoparasites could be found on *Avahi occidentalis* at any time of the year. *Lepilemur edwardsi* carried no lice, but mites and ticks, and both ectoparasite taxa were restricted to the rainy season. In the latter season, mites had a prevalence of 75% in *L. edwardsi* (*N* = 16 individuals). The difference in mite prevalence between the two lemur species in the rainy season was statistically significant (Fisher’s Exact Test: Chi sq. = 13.93, df = 1, *p* = 0.0002).

**Fig. 2:**
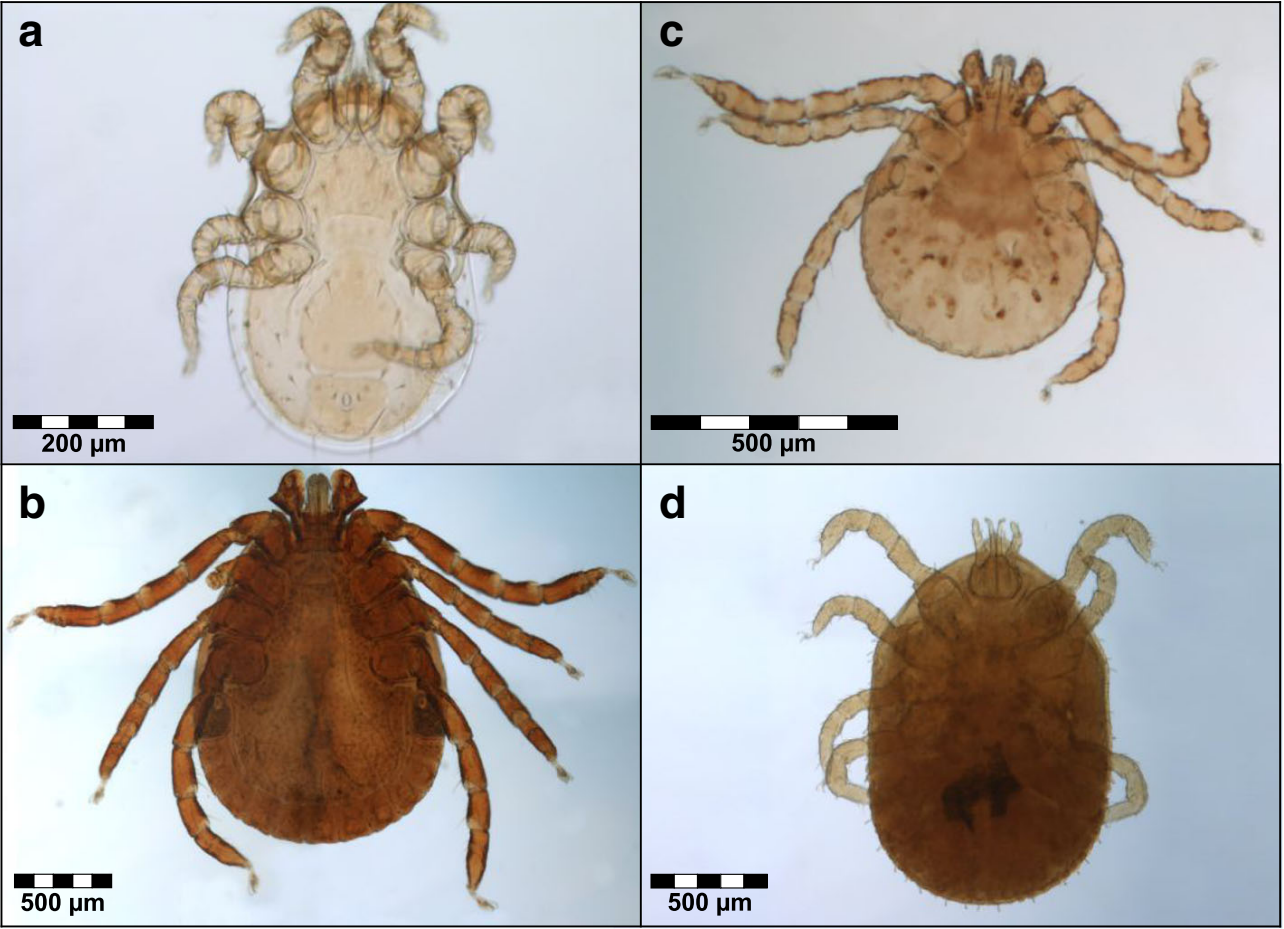
Ectoparasites collected from *Lepilemur edwardsi* at Ankarafantsika National Park in Madagascar. **a** *Aetholaps trilyssa*, **b** *Haemaphysalis lemuris* (adult male), **c** *H. lemuris* (larva) ** d** *Ornithodoros* sp. (nymph)

Mites on *L. edwardsi* were macroscopically visible, crawling through the animal’s fur all over the body. They were morphologically identified as *Aetholaelaps trilyssa* (Laelaptidae) (Fig. 2 a) [42]. Mite infestation was not associated with any evidence of skin alterations, such as alopecia, erythema or elevated desquamation.

Ticks were found in the inguinal region with a prevalence of 18.8% in *L. edwardsi* (*N* = 16 individuals) and were identified as *Haemaphysalis lemuris* (Ixodidae) (two larvae, one adult male, Fig. 2 b, c). They were only observed in hosts (*N* = 3 individuals) that were also infected with mites. Additionally, one of the host individuals was co-infected with *Ornithodoros* sp. (Argasidae) (one nymph, Fig. 2 d).

There was no significant difference in ectoparasite prevalence between males and females (Fisher’s Exact Test: Chi sq. = 0.76, df = 1, *p* = 0.38). Body mass of individuals infested with ectoparasites was not significantly different from those who were not (Mann-Whitney *U* = 51.5, n_1_ = 10, n_2_ = 14 *p* = 0.29).

### Hemoparasites

Microscopic examination of blood smears revealed filarial nematodes in both lemur species. The prevalence of microfilariae was 66.7% in *A. occidentalis* (*N* = 24 individuals) and 41.7% in *L. edwardsi* (*N* = 36 individuals). In both species, the prevalence was very similar across seasons (Table 1).

**Table 1:** Number of individuals infected with microfilariae (positives/total number of sampled animals)

Host species	Dry season	Rainy season
*A. occidentalis*	10/15 (66.67%)	6/9 (66.67%)
*L. edwardsi*	8/20 (40%)	7/16 (43.75%)

The GLMM revealed a significant difference in prevalence between the two host species (Table 2) with *A. occidentalis* showing a higher prevalence. Season, sex or time of blood collection had no significant effect on infection status, neither in *A. occidentalis* nor in *L. edwardsi* (Table 2).

**Table 2:** GLMMs testing the influence of the different factors on the probability of infection with microfilaria

Measure	Term	Estimate	Standard error	z	*p* value
Overall prevalence	Intercept	- 7.83	2.14	- 3.66	< 0.001*
	Species	16.15	3.61	4.48	< 0.0001*
Prevalence *A. occidentalis*	Intercept	- 0.16	1.19	- 0.13	0.890
	Sex	- 1.74	1.12	- 1.55	0.122
	Season	0.73	0.00	0.73	0.467
	Time of day	- 0.03	0.63	- 0.08	0.940
Prevalence *L. edwardsi*	Intercept	8.30	3.67	2.26	0.024*
	Sex	- 3.90	5.86	- 0.67	0.506
	Season	3.40	4.36	0.78	0.435
	Time of day	0.32	1.35	0.24	0.81

*Significant *p*-values (< 0.05)

In the positive samples, the number of microfilaremia (= intensity of infection) ranged from 0.1 to 27.9 microfilariae/μl with a mean of 3.1 ± 6.4 microfilariae/μl in *A. occidentalis* (*N* = 19 positive individuals) and from 0.2 to 10.1 microfilariae/μl with a mean of 2.4 ± 3.0 microfilariae/μl in *L. edwardsi* (*N* = 19 positive individuals) (Table 3).

**Table 3:** Microfilaria intensity in blood samples of *A. occidentalis* and *L. edwardsi*

Host species	Dry season	Rainy season
*A. occidentalis*	1.42 (± 1.88) mf/μl (*N* = 10, *n* = 10)	5.02 (± 9.03) mf/μl (*N* = 6, *n* = 9)
*L. edwardsi*	1.25 (± 1.67) mf/μl (*N* = 8, *n* = 10)	3.57 (± 3.73) mf/μl (*N* = 7, *n* = 9)

*mf* microfilariae

*N* number of sampled individuals

*n* number of positive samples

**Fig. 3:**
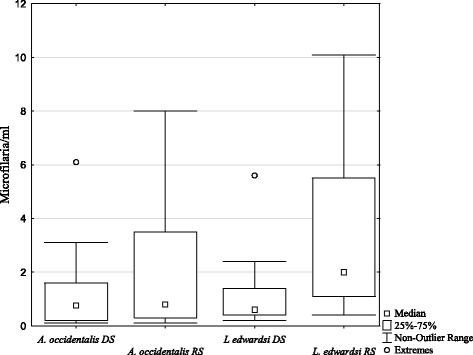
Microfilaria concentration in the blood of *A. occidentalis* and *L. edwardsi* in both seasons. DS = dry season, RS = rainy season (one outlier of 27.9 from *A. occidentalis* in the rainy season was removed)

The linear mixed effect model revealed no difference in microfilaremia between the two host species. In *L. edwardsi*, the factor season had the highest impact on microfilaremia. In contrast, season had no significant effect on microfilaremia in *A. occidentalis* (Fig. 3, Additional file 2)*.*

**Fig. 4:**
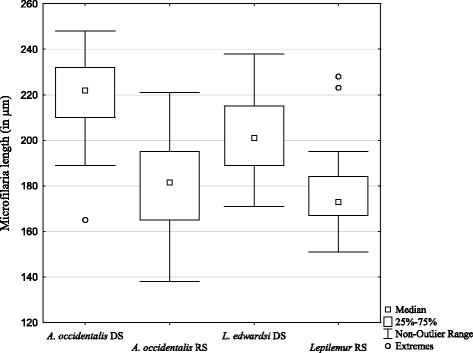
Microfilaria length of *A. occidentalis* and *L. edwardsi* in the dry and the rainy season. DS = dry season, RS = rainy season

On average, 8–9 microfilariae per sample were measured. Microfilariae of *A. occidentalis* were 200 ± 29.3 μm long (*N* = 100 microfilariae). Microfilariae of *L. edwardsi* were 190 ± 22.4 μm long (*N* = 100 microfilariae). This difference in length between microfilariae from the different host species was not statistically significant. However, microfilariae of both species were longer in the dry season than in the rainy season (Fig. 4) (Table 4).

**Table 4:** Mean length (± SD) of microfilariae in the two sampling seasons

Host species	Dry season	Rainy season
*A. occidentalis*	219 (± 17.9) μm (*N* = 7, *n* = 50)	181 (± 25.8) μm (*N* = 6, *n* = 50)
*L. edwardsi*	202 (± 19.5) μm (*N* = 6, *n* = 50)	177 (± 17.6) μm (*N* = 5, *n* = 50)

*N* number of sampled individuals

*n* number of measured microfilariae

The linear mixed effect model found microfilaria in *L. edwardsi* to be significantly longer in the dry than in the rainy season (*p* = 0.024, Additional file 3). In *A. occidentalis*, this factor was not significant in the full model, but a model with the single factor season explained the results better than the null model (L. Ratio: 8.47, df = 5, *p* = 0.004). The seasonal difference in length that is clearly visible at least in *L. edwardsi* might indicate the presence of different filarial species at different times of the year. In both host species body mass of individuals infected with microfilaria was not significantly different from those who were not (*L. edwardsi*: Mann-Whitney *U* = 53.0, n_1_ = 15, n_2_ = 9 *p* = 0.41; *A. occidentalis* Mann-Whitney *U* = 42.0, n_1_ = 8, n_2_ = 12, *p* = 0.68).

Co-infections with ectoparasites and microfilaria could be observed in *L. edwardsi* (*n* = 6). However, there were also individuals, which were infested with ectoparasites but did not show microfilariae (*n* = 6) or were infected with microfilariae but did not show ectoparasites (*n* = 4).

The ITS1–5.8S–ITS2 rDNA region of microfilariae from a blood sample of *L. edwardsi* could be successfully amplified. The sequence with a length of 1363 bp showed 83–96% identity to different nematodes belonging to the family Onchocercidae such as *Onchocerca* sp., *Brugia* sp. and *Mansonella* sp. However, the percentage of similarity did not allow reliable genus assignment. The top hit was an unnamed filarial nematode, which had been found in another lemur species, the Verreaux’s sifaka (*Propithecus verreauxi*) (query coverage: 47%, identity: 96%, E-value: 0; GenBank accession no.: LN869520) [43].

## Discussion

We examined whether host sleeping site ecology shapes the pattern of parasitism with regard to infection with ectoparasites and vector-borne hemoparasites by studying two ecologically similar sympatric lemur species, *Avahi occidentalis* and *Lepilemur edwardsi*. In general ectoparasite infestation was low compared to other mammals. Three species of ectoparasites, one mite and two tick species, were collected from *L. edwardsi*, whereas no ectoparasites were found on *A. occidentalis*. *L. edwardsi* showed infestation with ectoparasites only during the wet season*.* On the other hand, prevalence of microfilaria was significantly higher in *A. occidentalis* than in *L. edwardsi*. Neither host species showed a difference in body mass between individuals carrying mites, ticks or microfilaria and those who did not. This might be an indication that the animals’ health and most likely fitness are not affected by the low parasite intensity with ecto- and haemoparasites. However, some individuals with a low body condition may have deceased quickly or fell victim to predators and may therefore not have been detected. Moreover, ectoparasites may also transmit vector-born diseases such as borreliosis [44] which may lead to rapid illness and mortality before being detected in differences in body weight. The absence of ectoparasites in the openly sleeping host *A. occidentalis* and their seasonal presence in the tree hole-sleeping *L. edwardsi* support the hypothesis that tree-holes may constitute a suitable habitat for ectoparasites. *Aetholaelaps trilyssa*, a mite belonging to the family Laelaptidae, showed a prevalence of 75% in *L. edwardsi*. Mites of this family are commonly found on various lemur species but were never associated with clinical disease [45, 46]. Some laelaptid mites are known to be nidicolous temporary parasites, living in the nest of the host but infesting the host for feeding [47]. Apart from the morphological description, there is not much known about the Malagasy endemic genus *Aetholaelaps*, but it is possible that *Aetholaelaps trilyssa* behaves as a nidicolous temporary mite. In that case, this mite’s ecology would explain why it was found at high prevalence in *L. edwardsi*, a host that sleeps in regularly revisited tree holes, but not in *A. occidentalis*, a species that sleeps on open branches and rotates its sleeping site more frequently [35, 36]. The fact that some host species adapt their sleeping behavior by changing their sleeping sites in order to avoid ectoparasite infestation supports this explanation [16, 48].

A dark, moist and regularly re-visited tree hole may favor parasitism by ticks, especially with nest-adapted tick species. *Ixodes hexagonus*, for instance, is often found in or around nests of hosts and is often associated with nesting mammals like hedgehogs [49]. Ixodid ticks were observed on three *L. edwardsi* individuals (18.8%) and identified as *Haemaphysalis lemuris* (larva and adults), which has previously been described as parasitizing *Lepilemur* spp. [4, 50, 51]. Furthermore, one of these three individuals was co-infected with *Ornithodoros* sp., an argasid tick. To our knowledge, argasid ticks have not been documented to parasitize lemurs before.

Ticks are known to parasitize several host species and *H. lemuris*, in particular, has been found to parasitize a large variety of lemur species (*Microcebus rufus, M. griseorufus, Lemur catta, Varecia variegata, Lepilemur ruficaudatus, L. leucopus, Propithecus verrauxi*), some of which sleep in tree holes and some openly [52–54]. Given that many lemur species with a similar ecology were reported to be parasitized by this ectoparasite, its lack on *A. occidentalis* was surprising. However, it has to be kept in mind that only a relatively small number of individuals was sampled, and infestation may therefore have remained unnoticed if the overall prevalence was low. Nevertheless, the sampled individuals in this study nearly represent the whole *Lepilemur* and *Avahi* population of the 30.6 ha study site [55, 56]. In general, the number of studies investigating ectoparasitism in *Avahi* spp. is very limited, and so far only the sucking louse *Phtirpediculus avahidis* has been described to parasitize this genus of lemurs [57]. In summary, the pattern of ectoparasitism found in these two lemur species suggests that the choice of sleeping sites may drive the presence of ectoparasites in these primates. However, the benefits provided by tree holes, e.g. protection from predators and insulation [48], probably outweigh the costs that may be imposed by the elevated ectoparasite infestations.

All ectoparasites were only observed in the rainy season, indicating annual dynamics of parasite activity. Survival and development of mites and ticks is directly influenced by temperature, i.e. occurrence and abundance of ticks often increases with high temperatures and after rainfall [3, 58]. The tick *Amblyomma variegatum*, for instance, was even observed to disappear in cattle in the dry season [59]. As for mites, they have repeatedly been documented with invariable prevalence and intensity at different times of the year [60, 61]. Nevertheless, mites were also noted to reproduce more intensively during the reproductive periods of their hosts, i.e. while they were pregnant or lactating. As a consequence, the annual cycle may be influenced by seasonal abiotic conditions as well as seasonal reproductive activities of the host [62]. The pregnancy period of *L. edwardsi* in Ankarafantsika National Park lasts from July to November and lactation starts in October, coinciding with the beginning of the rainy season [63]. In this study, the samples representing the rainy season were collected in March and April, corresponding to the end of the rainy season. No data was collected at the beginning of the rainy season due to heavy rainfalls. However, one could speculate that the mites collected from *L. edwardsi* in March and April are the remnants of a preceding reproductive peak of the mite *A. trilyssa*, which might have occurred during the lactation period at the beginning of the rainy season.

Filarial nematodes were detected microscopically in blood smears of both lemur species. Length measurements revealed that microfilariae of both lemur species were significantly longer in the dry than in the rainy season. This might indicate the presence of two different nematode species, one occurring predominantly in the dry, and the other in the rainy season. So far, four species of microfilariae were described in lemurs, all belonging to the family Onchocercidae [64]. Of these species, the microfilariae found in the dry season correspond in size to *Paulianfilaria pauliani*. However, different methods of blood smear fixation can result in various microfilaria lengths, so that species identification by comparison of microfilariae measurements from different studies alone becomes unreliable [21]. No adult worm could be recovered as dissections of animals would have been necessary. Thus, further morphological identification was not feasible. Unfortunately, only microfilaria DNA from one sample of *L. edwardsi*, taken in the rainy season, could be successfully sequenced. The generated sequence was most similar to sequences derived from microfilariae found in Verreaux’s sifakas (*Propithecus verreauxi*), belonging to the family Onchocercidae with no further assigned name [43]. To date, these are the only two sequences from Malagasy filarial nematodes which are available in the NCBI GenBank. Consequently, BLAST search did not enable genus allocation. No RNAlater-samples were taken in the dry season, so that microfilariae detected in the dry season samples could only be analyzed morphologically.

It is likely that the filarial nematode species have been transmitted by different arthropod vectors which may vary in their abundance between the dry and the rainy season. The vectors of these microfilariae are most likely mosquitoes or other flying insects rather than ticks or lice, since microfilariae were also found in *A. occidentalis*, who did not show any ectoparasites [65, 66]. It was hypothesized that the tree hole-sleeping *L. edwardsi* should contain microfilariae less often than *A. occidentalis* that sleeps openly. In support of the hypothesis that tree holes may confer some degree of protection from flying vectors, such as mosquitoes, the prevalence of microfilariae was significantly higher in *A. occidentalis* than in *L. edwardsi*. A total of 66.7% of the population of *A. occidentalis* carried microfilariae, whereas only 41.7% of *L. edwardsi* were infected. As the lifestyles of these two lemur hosts are very similar in terms of habitat use and group size and they are also comparable in body size, other factors are less likely to cause this difference in filarial prevalence. However, it is possible that the microfilaria species may be better adapted to *A. occidentalis*, enhancing microfilaria survival in this primate host. Furthermore, *A. occidentalis* may be more susceptible to microfilarial infection. Another aspect to be considered is that the two lemur species may carry different microfilaria species, one of which occurring more frequently, explaining the difference in prevalence. In case we are dealing with only one filarial species, which infects both lemur species, *A. occidentalis* might be more susceptible. The findings also suggest that the insect vectors are diurnal, since both lemur species stay in their sleeping sites during daytime and differ then in exposure, whereas they are both nocturnal arboreal foragers. However, no difference in the level of microfilaremia was detected between the two hosts. Nevertheless, these findings may indicate that sleeping in tree holes may affect prevalence of hemoparasites in primate hosts.

In contrast, no effect of season on microfilaria prevalence was found despite presumably higher vector abundance during the wet season, neither in *A. occidentalis* nor in *L. edwardsi*. Filarial nematodes have a long prepatent period and once developed, they persist in their host for several months, possibly concealing a seasonal effect. In Kirindy Forest, a habitat with comparable seasonal conditions to those in Ankarafantsika National Park, Verreaux’s sifakas (*Propithecus verreauxi*) hosting microfilaria also showed no effect of seasonality on infection [43]. However, when looking at the level of microfilaremia as a proxy for infection intensity, the model containing the factor season in *L. edwardsi* differed significantly from the null model, indicating higher infection intensity in the rainy season (cf. Fig. 3).

No relation was detected between sex and either presence or infection intensity of filarial nematodes. Some studies have reported higher prevalence in males, e.g. in raccoons (*Procyon lotor*) [67], probably caused by sex-differences in body size or in hormone levels, such as immunosuppressive effects of testosterone [68]. The studied lemur species do not display sexual dimorphism in size, resulting in equal exposure of both sexes to vectors. Additionally both species are pair-living with dominant or at least co-dominant females [34], which may result in a smaller difference in androgen levels between sexes compared to other mammals [69]. Males and females may therefore be equally immunocompetent, although future studies are needed to investigate this hypothesis in more detail.

Since the presence of microfilariae circulating in the blood stream is subjected to a circadian rhythm [70], time of sampling was included as a factor in the statistical models. However, neither the likelihood nor intensity of infection was affected by the time of blood collection. Microfilaraemia levels are dependent on the intravascular distribution of the parasite. Dreyer et al. [71] documented an average microfilaria concentration that was 1.25 times higher in capillary blood than in venous blood obtained at the same time. Moreover, the number of microfilariae was proportionally higher in the capillary system of the skin at the time when biting activity of the local mosquito vector was highest. In our study, blood smears were sometimes derived from venous blood drawn from the femoral vein and at other times from capillary blood that was collected in the process of ear-marking and tissue collection. This sampling disparity might have led to the absence of an effect of time of blood collection on microfilaria concentration, as well as to the lack of difference in infection intensity between *A. occidentalis* and *L. edwardsi* mentioned above.

## Conclusions

The findings of the present study support the hypothesis that sleeping site ecology affect patterns of parasitism in nocturnal, arboreal primate hosts in a seasonal environment. *L. edwardsi*, which uses tree holes as sleeping sites, showed infestation with three different ectoparasite taxa during the wet season, whereas no ectoparasites were found on *A. occidentalis*, which sleeps on open branches but is otherwise ecologically similar. Thus, repeatedly revisited tree holes seem to present a driver of ectoparasite infestations. In contrast, prevalence of microfilaria was significantly higher in *A. occidentalis* than in *L. edwardsi*. Hence, sleeping in tree holes might protect from bites of flying insects that transmit hemoparasites, such as filarial nematodes. In conclusion, sleeping site ecology is an important ecological driver of parasite distribution and transmission in wild populations.

## Additional files

Additional file 1: Table with the individual blood sampling frequency in the dry and in the rainy season. (DOCX 15 kb)

Additional file 2: Table with the results of the LMMs testing the influence of host species, sex, season and time of day on the number of microfilaremia (= intensity of infection). (DOCX 17 kb)

Additional file 3: Table with the results of the LMMs testing the influence of host species, sex and season on microfilaria length. (DOCX 18 kb)
