# Supplementary material for: Sleeping site ecology, but not sex, affect ecto- and hemoparasite risk, in sympatric, arboreal primates (Avahi occidentalis and Lepilemur edwardsi)
Source: Front Zool. 2017 Sep 20;14:44. doi: 10.1186/s12983-017-0228-7 (PMC5607495; doi:10.1186/s12983-017-0228-7)
Supplement: Supplementary file 1 — Table with the individual blood sampling frequency in the dry and in the rainy season. (DOCX 15 kb) [file 12983_2017_228_MOESM1_ESM.docx]

Additional File 1: Individual blood sampling frequency in the dry and in the rainy season

| Species | Anima ID | Dry season | Rainy season |
| --- | --- | --- | --- |
| *L. edwardsi* | L0113 | 0 | 1 |
| *L. edwardsi* | L0213 | 1 | 1 |
| *L. edwardsi* | L0313 | 2 | 1 |
| *L. edwardsi* | L0413 | 1 | 1 |
| *L. edwardsi* | L0513 | 1 | 0 |
| *L. edwardsi* | L0613 | 1 | 0 |
| *L. edwardsi* | L0713 | 1 | 1 |
| *L. edwardsi* | L0813 | 1 | 0 |
| *L. edwardsi* | L0913 | 1 | 1 |
| *L. edwardsi* | L1013 | 2 | 1 |
| *L. edwardsi* | L1113 | 2 | 0 |
| *L. edwardsi* | L1213 | 2 | 1 |
| *L. edwardsi* | L1313 | 1 | 0 |
| *L. edwardsi* | L1413 | 2 | 0 |
| *L. edwardsi* | L1513 | 2 | 1 |
| *L. edwardsi* | L1613 | 1 | 0 |
| *L. edwardsi* | L1713 | 1 | 0 |
| *L. edwardsi* | L1813 | 1 | 0 |
| *L. edwardsi* | L1913 | 1 | 2 |
| *L. edwardsi* | L2013 | 1 | 2 |
| *L. edwardsi* | L2113 | 1 | 0 |
| *L. edwardsi* | L2213 | 1 | 1 |
| *L. edwardsi* | L0214 | 0 | 1 |
| *L. edwardsi* | L0314 | 0 | 1 |
| *L. edwardsi* | L0414 | 0 | 1 |
| *A. occidentalis* | A0113 | 1 | 0 |
| *A. occidentalis* | A0213 | 1 | 1 |
| *A. occidentalis* | A0313 | 2 | 0 |
| *A. occidentalis* | A0413 | 1 | 0 |
| *A. occidentalis* | A0513 | 1 | 0 |
| *A. occidentalis* | A0613 | 1 | 0 |
| *A. occidentalis* | A0713 | 1 | 0 |
| *A. occidentalis* | A0813 | 1 | 0 |
| *A. occidentalis* | A0913 | 1 | 0 |
| *A. occidentalis* | A1013 | 1 | 0 |
| *A. occidentalis* | A1113 | 1 | 2 |
| *A. occidentalis* | A1213 | 1 | 0 |
| *A. occidentalis* | A1313 | 1 | 1 |
| *A. occidentalis* | A1413 | 1 | 0 |
| *A. occidentalis* | A1513 | 1 | 0 |
| *A. occidentalis* | A1813 | 1 | 0 |
| *A. occidentalis* | A0114 | 0 | 2 |
| *A. occidentalis* | A0214 | 0 | 2 |
| *A. occidentalis* | A0314 | 0 | 2 |
| *A. occidentalis* | A0614 | 0 | 1 |
| *A. occidentalis* | A0714 | 0 | 1 |
