# Supplementary material for: Sleeping site ecology, but not sex, affect ecto- and hemoparasite risk, in sympatric, arboreal primates (Avahi occidentalis and Lepilemur edwardsi)
Source: Front Zool. 2017 Sep 20;14:44. doi: 10.1186/s12983-017-0228-7 (PMC5607495; doi:10.1186/s12983-017-0228-7)
Supplement: Supplementary file 2 — Table with the results of the LMMs testing the influence of host species, sex, season and time of day on the number of microfilaremia (= intensity of infection). (DOCX 17 kb) [file 12983_2017_228_MOESM2_ESM.docx]

Additional file 2: Results of the LMMs testing the influence of host species, sex, season and time of day on the number of microfilaremia (= intensity of infection).

| Measure | Term | Value | Standard error | t-value | *p* value |
| --- | --- | --- | --- | --- | --- |
| Microfilaremia | Intercept | - 0.19 | 0.35 | - 0.54 | 0.600 |
|  | Species | 0.32 | 0.52 | 0.62 | 0.543 |
| Microfilaremia *A. occidentalis* | Intercept | 1.09 | 1.15 | 0.95 | 0.363 |
|  | Sex | 0.32 | 0.86 | 0.37 | 0.716 |
|  | Season | - 0.68 | 0.71 | - 0.96 | 0.438 |
|  | Time of day | - 1.18 | 0.92 | - 1.28 | 0.330 |
| Microfilaremia *L. edwardsi* | Intercept | 2.20 | 0.90 | 2.45 | 0.040 |
|  | Sex | - 0.96 | 0.58 | - 1.66 | 0.137 |
|  | Season | - 0.97 | 0.51 | - 1.91 | 0.099 |
|  | Time of day | - 0.38 | 0.29 | - 1.29 | 0.240 |
