# Supplementary material for: Sleeping site ecology, but not sex, affect ecto- and hemoparasite risk, in sympatric, arboreal primates (Avahi occidentalis and Lepilemur edwardsi)
Source: Front Zool. 2017 Sep 20;14:44. doi: 10.1186/s12983-017-0228-7 (PMC5607495; doi:10.1186/s12983-017-0228-7)
Supplement: Supplementary file 3 — Table with the results of the LMMs testing the influence of host species, sex and season on microfilaria length. (DOCX 18 kb) [file 12983_2017_228_MOESM3_ESM.docx]

| Measure | Term | Value | Standard error | t-value | *p* value |
| --- | --- | --- | --- | --- | --- |
| Length | Intercept | 199.20 | 5.31 | 37.51 | < 0.0001* |
|  | Species | - 7.27 | 8.26 | - 0.88 | 0.392 |
| Length *A. occidentalis* | Intercept | 183.92 | 7.99 | 22.99 | < 0.0001* |
|  | Sex | - 2.25 | 9.60 | 3.61 | 0.819 |
|  | Season | 32.61 | 9.03 | 0.82 | 0.172 |
| Length *L. edwardsi* | Intercept | 174.72 | 7.51 | 23.26 | < 0.0001* |
|  | Sex | 4.62 | 7.24 | 0.64 | 0.551 |
|  | **Season** | **26.26** | **6.13** | **4.27** | **0.024*** |

Additional file 3: Results of the LMMs testing the influence of host species, sex and season on microfilaria length.

* Significant p-values (> 0.05)
